# Parasitic etiology of diarrhea and associated factors among under-five-year children attending Mizan-Tepi University Teaching Hospital, Southwest Ethiopia

**DOI:** 10.11604/pamj.2023.45.187.38263

**Published:** 2023-08-30

**Authors:** Teshale Worku, Teka Haile, Samuel Sahile, Tadesse Duguma

**Affiliations:** 1 College of Medicine and Health Sciences, Mizan-Tepi University, Mizan-Aman, Ethiopia

**Keywords:** Diarrhea, parasitic etiology, under-five-year children, Southwest Ethiopia

## Abstract

**Introduction:**

globally, an estimated two billion cases of diarrhea occur every year, and an estimated 1.7 million children under the age of five years, mostly in developing countries die due to diarrheal cases. It is caused by different enteropathogens such as bacteria, viruses, and parasites. Diarrhea caused by parasitic etiology is common in under-five-year children in sub-Saharan Africa. The objective was to investigate the parasitic etiology of diarrhea among under-five-year children in Mizan-Tepi University Teaching Hospital, Southwest Ethiopia.

**Methods:**

institution-based cross-sectional study was conducted from September to November 2021. A total of 300 under-five-year children presenting with diarrhea in Mizan-Tepi University Teaching Hospital were included in the study. Data used to assess associated factors for intestinal parasitic infections (IPIs) was collected using a structured questionnaire. Stool specimens were collected from the study participants for investigations of the parasitic etiology of diarrhea. The specimens were processed microscopically using direct wet mount and formol ether concentration techniques. Modified Ziehl-Neelsen staining of formol-ether concentrated specimens was also performed. Data was entered using Epi-Data version 4.6.0 and analyzed using Stata/SE version 14.0. Crude odds ratio and adjusted odds ratio were used to determine the association.

**Results:**

from a total of 300 children who participated in this study, 68 (22.67%) were positive for at least one intestinal parasite. E. histolytica23(7.67%) and G. lamblia17(5.67%) were the predominant parasitic etiologies and 28(9.33%) were positive for intestinal helminthic parasites; 11(3.67%) Ascaris lumbricoides, 10(3.33%) Trichuris trichiura, 4(1.33%) Hymenolepsis nana, and 3(1%) were double infection with Trichuris trichuria and Ascaris lumbricoides. Multivariable analysis revealed the age group category 2-3 years of age children was more attributable to intestinal parasitic infections (AOR= 0.466, 95% CI: 0.204-0.976).

**Conclusion:**

the overall prevalence of intestinal parasitic infections (IPIs) in this study was 22.67%. Diarrheal etiology of intestinal parasites among under five years of children identified in this study were significantly associated with maternal/ guardians´ educational status, (AOR=94.900, 95% CI: 24.664-365.155) use of unprotected water for drinking, (AOR =25.189, 95% CI: 4.671-135.847).

## Introduction

Globally, about two billion cases of diarrhea occur every year [1]. It is caused by bacteria, viruses, protozoa, and helminths [2]. In developing countries, diarrhea is more commonly caused by intestinal parasites (IPs) and bacterial pathogens than by viruses [3]. These etiologies are transmitted through contaminated hands, inanimate objects, and mechanical vectors [4]. Diarrhea kills more under-five-year children than Acquired Immunodeficiency Syndrome (AIDS), malaria, and measles in combination can, indicating the severe impact of the disease in this age group [5]. Around 90% of morbidity and mortality occurred in South Asia and sub-Saharan African countries, which are more attributable to unsafe water, inadequate sanitation, and insufficient hygiene [3,6]. It causes one in eight deaths among under-five-year children and more than 5,000 children continue to die each year [7]. In Ethiopia, the prevalence of diarrhea among under-five-year children was 12.1% [8]. Studies carried out in different localities of Ethiopia revealed a high burden of diarrhea. For instance, 22.1% in rural areas of the North Gondar Zone, 28.9% in Nekemte Town of western Ethiopia, 27.3% in the Jigjiga District of the Somali Region, and 22.5% in the Kersa District of Eastern Ethiopia [9,10]. As in other parts of Ethiopia, diarrhea among under-five-year children is a common public health problem in the southern parts of Ethiopia. It is one of the most common reasons for children to visit health institutions. Most of the time, diarrheal cases were managed clinically rather than through laboratory-based identification of the causative agents of the illness. Hence, there is a lack of complete data to document its burden due to inadequate definitive diagnosis. Several studies indicated that the educational status of parents, occupation, income, contaminated water source, duration of breastfeeding, failure to wash hands adequately, inappropriate use of latrines and age of the child were significant forecasters of diarrhea among under-five-year children [11-14]. This study aims to identify the parasitic etiology of diarrhea and associated factors among under-five-year children attending Mizan-Tepi University Teaching Hospital, Southwest Ethiopia.

## Methods

**Study type and design**: an institution-based cross-sectional study was conducted from September to November 2021 at Mizan-Tepi University Teaching Hospital.

**Study setting**: this study was carried out in Mizan-Tepi University Teaching Hospital which is located in the Aman sub-city of Mizan Aman City, which is an administrative city of Bench-Sheko Zone. The Zone is found in Southwest Ethiopia Regional State.

**Study participants**: randomly selected under-five-year children attending Mizan-Tepi University Teaching Hospital Pediatric Outpatient Department (POPD) presenting with diarrhea were included in the study.

**Inclusion criteria**: all under-five children with diarrhea who visits POPD in Mizan-Tepi- University Teaching Hospital were included in our study.

**Exclusion criteria**: children who were on Medication during the time of sample collection were excluded from the study.

**Sample size and sampling procedure**: the sample size was determined using single population formula considering the following assumptions: Z Þ/2 at 95% confidence interval, proportion of under-five children with diarrhea who had parasitic agents of infection was taken 26.6% from previous similar study conducted in Hawassa town, Southern, Ethiopia [5] and margin of error (d) of 5% were used. Adding a 10% non-response rate, the final sample size was collected to be = 330.

**Sampling technique**: simple random sampling technique was used to select the under-five children who attended at Mizan-Tepi University Teaching Hospital who experienced diarrheas during the study period were included. The researchers selected a subset of participants from a population randomly. Each member of the population has an equal chance of being selected. Data is then collected from as large a percentage as possible of this random subset using lottery method to incorporate all the study participants until the sample size reached which was determined by assigning sequential values to each participant within a population, then randomly selecting those values.

**Methods of collecting data and instrumentation**: a pre-tested structured questionnaire was used to collect the demographic data. The questionnaire includes socio-demographic, environmental, and behavioral factors for the parasitic etiology of diarrhea. The questionnaire was developed in English and then translated into the local language, Amharic. Back translation from Amharic to English language was done by another person who had good skills in the two languages to check for its consistency. Pre-testing of the questionnaires was done on 5% of the participants at another hospital a week before the actual data collection period. Interviewer-filled information was obtained from each study participant after taking written informed consent from their parents.

**Sample collection and processing**: about 2 grams of diarrheic stool samples were collected from the participants in screw-capped containers. The samples were documented with the necessary client information and transported to the parasitology laboratory within 30 minutes of collection. In the laboratory, the direct saline wet mount was performed and observed for the detection and identification of motile and non-motile intestinal parasites. The concentration technique was also conducted according to the standard procedures for the detection of intestinal parasites found in small numbers. The preparations were examined microscopically using a low-power objective as well as the 40x objective to examine small cysts and eggs. Different staining techniques such as iodine stain and modified Ziehl-Neelsen Staining (ZNS) method were also performed following concentration by the formol ether to detect oocyst stages of protozoans.

**Data quality assurance**: the questionnaire was checked and pre-tested before conducting data collection. Data was collected by using a local language that is being spoken by respondents. During the collection of data, the participants were informed about the research to prevent confusion and information bias. Data collection by questionnaire, stool processing, and stool sample collection was done by following the standard operating procedure, pre-analytic, analytic, and post-analytic phases of quality assurance were applied. Samples were checked for mixing of stool with urine. The reagents were checked for consistency and expiration date. Examination slides were prepared in duplicates and each slide was examined by two investigators.

**Methods of data analysis**: data was entered using Epi-Data version 4.6.0 and then exported into Stata version 14.0 analysis. Descriptive statistics parameters such as frequency and mean were computed. Crude odd ratios (COR) and adjusted odds ratios (AOR) were calculated to check the association between the variables. P-value <0.05 was considered for the statistically significant association. Finally, the magnitude of association between different variables with the outcome variable was measured by adjusted odds ratio with a 95% confidence interval.

**Availability of data and materials**: the data are available from the corresponding author upon reasonable request.

**Funding**: this study has not received any funds.

**Ethics approval and consent to participate**: ethical clearance was obtained from the College of Medicine and Health Sciences review committee. After approval, a formal letter was sent to the administrator of Mizan-Tepi University Teaching Hospital to get permission for the study. Communication was made between the administrator and the head of the ward before the commencement of the study. Written informed consent was obtained from each study participant.

## Results

**Figure 1:**
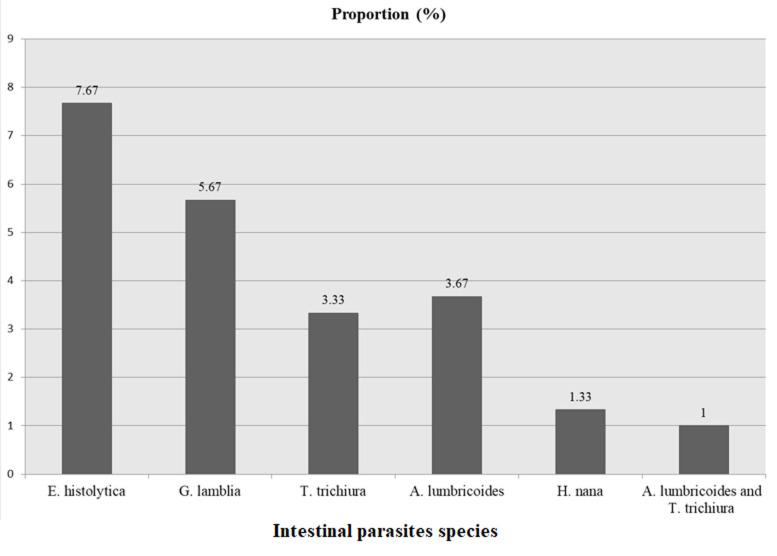
intestinal parasites species distribution among under-five children who attended Mizan-Tepi University Teaching Hospital

**Socio-demographic characteristics of the participants**: the distribution of age, sex, and residence of the participants was presented below (Tableau 1). A total of 300 children aged from six months to five years presenting with diarrhea to Mizan-Tepi University Teaching Hospital participated in the study. The majority of the children, 145(48.33%) were aged below two years. The remaining 76(25.33%) and 79(26.33%) were children aged between two to three and three up to five years respectively. One hundred twenty-three (41%) were male and the remaining 177(59%) were female. Regarding the residence of the participants, 150(50%) urban (Mizan Aman town) and 150(50%) rural resident study participants were participated in the study. **Prevalence of intestinal parasites**: a total of 300 under-five children were participated in this study. Diarrheic stools were taken from each participant and the specimens were first characterized macroscopically. Accordingly, more than half of the children 221(73.67%) had watery diarrhea, 77 (25.67%) presented with mucoid diarrhea, and only 2(0.67%) children had bloody diarrhea. Of the total under-five diarrheic children, 68(22.67%) were positive for at least one intestinal parasite species. As indicated above (Figure 1), *Entamoeba histolytica (E. histolytica)* was the predominant parasite accounting for 23(7.67%) followed by *Giardia lamblia (G. lamblia)* 17(5.67%). From intestinal helminths, 11(3.67%) *Ascaris lumbricoides (A. lumbricoides)*, 10(3.33%) *Trichuris trichiura (T. trichiura)*, 4(1.33%), and *Hymenolepsis nana (H. nana)* were detected. Mixed infection with *T. trichiura* and *A. lumbricoides* was also detected in 3(1%) children.

**Table 1:** socio-demographic characteristics of under-five-year children with diarrhea in Mizan-Tepi University Teaching Hospital

Socio-demographic variables	Category	Frequency
**Age in years**	< 2 years	145(48.33%)
	2-3 years	76(25.33%)
	Above 3-5 years	79(26.33%)
**Sex**	Male	123(41%)
	Female	177(59%)
**Residence**	Urban	150(50%)
	Rural	150(50%)

**Factors associated with intestinal parasitic infections**: Socio-demographic, environmental, and behavioral factors associated with intestinal parasitic infections among the participants were presented below (Tableau 2 and Tableau 3). There is no statistically significant difference in the prevalence of intestinal parasitic infections to sex was observed (p > 0.05). The prevalence of intestinal parasites among the children from the rural area and urban areas was 16.67% and 6% respectively with a statistically significant difference (COR= 0.248, 95% CI: 0.135-0.455), AOR=4.028, 95% CI: 2.045-7.932). The prevalence of intestinal parasitic infection among children aged less than two years was 35(11.67%) and that of two to three years was 19(6.33%), and above three years was 14(4.67%). The multivariable analysis revealed that the age group between 2-3 years was a predictor of intestinal parasitic infection showing significant difference in the prevalence of intestinal parasites (AOR=0.466, 95% CI: 0.204-0.976). The prevalence of intestinal parasites concerning the number of children and the educational status of mothers was significantly observed (Tableau 3). From the assessed environmental factors, children who use unprotected water for drinking (AOR, 25.189, 95% CI: 4.671-135.847) were more infected with intestinal parasites (p <0.05). There was no a statistically significant difference in intestinal parasitic infection (IPIs) among under five-year children who did not wash their hands (14.67%) and who practice hand washing (7.33%), those who wash fruits before meal (3.67%) and their counterparts (18.33%) (Table 3). The rate of IPIs among the subjects who use pipe water for drinking was 1.33% which showed a significant difference to those who use unprotected spring (3%) with AOR (25.189, 95% CI: 4.671-135.847). There was increased prevalence of intestinal parasitic infection among the subjects who use untreated water (20%) than who use treated water (2%) (COR = 0.270, 95% CI: 0.093-0.782), AOR=2.559, 95% CI: 0.386-1.307). Intestinal parasitic infection among who have proper waste disposal sites was 2.33% than those who did not have (19.67%).

**Table 2:** univariate and multivariable analyses of socio-demographic factors with intestinal parasitic infections among under-five-year children who attended Mizan-Tepi University Teaching Hospital

Variable	Category	Intestinal parasites				COR (95% CI)	AOR (95% CI)
		**Positive**		**Negative**			
		No	%	No	%		
**Age in years**	< 2 years	35	24.13	110	75.87	1	1
	2-3 years	19	25	57	75	0.955(0.502,1.817)	0.466(0.204,0.976)*
	> 3 years	14	17.72	65	82.28	1.77(0.740,2.950)	0.4639(0.195,1.102)
**Sex**	Male	26	21.14	97	78.86	1	1
	Female	42	23.73	135	76.27	0.795(0.455,1.388)	1.162(0.599, 2.254)
**Residence**	Urban	18	12	132	88	1	1
	Rural	50	33.33	100	66.67	0.248(0.135,0.455)	4.028(2.045,7.932) *
**Number of Children**	< 3	17	8.1	194	91.9	1	1
	> 3	51	57.3	37	42.7	0.065(0.034,0.041)	18.264(9.554,34.915)
**Maternal educational status**	No formal education	27	81.82	6	18.18	0.011(0.003,0.041)	94.900(24.664,365.155)*
	Read & write	19	50	19	50	0.113(0.028,0.463)	8.848(2.160,36.240) *
	Primary	14	12.84	95	87.16	0.290(0.083,1.018)	3.449(0.982,12.106)
	Secondary and/ or above	8	6.56	114	93.44	1	1

**COR** (Crude odds ratio), **AOR** (Adjusted odds ratio), **1** represents the reference category, **CI**= Confidence interval * stands for P< 0.05

**Table 3:** univariate and multivariable analyses of environmental and behavioral factors with intestinal parasitic infection among under-five year children in Mizan-Tepi University Teaching Hospital

Environmental factor	Observation	Result of stool examination				COR (95% CI)	AOR (95% CI)
		**Positive**		**Negative**			
		**No**	**%**	**No**	**%**		
Availability of toilet room	Yes	53	18.86	228	81.14	1	1
	No	13	68.42	6	31.58	0.112(0.041, 0.309)	0.924(0.136,6.271)
Hand washing after toilet	Yes	22	10.05	197	89.95	1	1
	No	44	54.32	37	45.68	0.094(0.051, 0.174)	0.691(0.140,3.422)
Fruit washing Before meal	Yes	11	5.50	189	94.50	1	1
	No	55	55	45	45	0.050(0.025, 0.101)	2.631(0.573,12.081)
Water source for Drinking	Pipe water	4	3.80	105	96.20	1	1
	Protected spring	53	29.28	128	70.71	0.270(0.093, 0.782)	2.559(0.386,1.307)
	Unprotected spring	9	90	1	10	0.003(0.001,0.012)	25.189(4.671,135.847)*
Treating water before drunk	Yes	6	3.26	178	96.74	1	1
	No	60	51.72	56	48.28	0.029 (0.012,0.072)	3.553(0.814,15.507)
Proper waste disposal site	Yes	7	3.10	219	96.90	1	1
	No	59	79.73	15	20.27	0.005(0.002, 0.013)	4.471(0.211,93.252)

**COR** (Crude odds ratio), **AOR** (Adjusted odds ratio), **1** represent the reference category, **CI**= Confidence interval * stands for P< 0.05

## Discussion

The overall prevalence of intestinal parasitic infections (IPIs) in our study was 22.67%. Different parasitic causes of diarrhea among under-five-years of children identified in this study were also reported by several studies conducted elsewhere [15,16]. The prevalence of IPIs found in this study was consistent with that reported from Hawassa, Southern Ethiopia (26.6%) [3] and lower than that reported from Addis Ababa (27.5%) [16], Yirgalem, Southern Ethiopia (49.56%) [17], Bale, Eastern Ethiopia (38.5%) [18] and other countries such as Nigeria (23.3%) [19] Bangladesh (70%) [20], Cameroon (59.2%) [21], India (46.5%) [22], and Gabon (61%) [23]. On the other hand, the overall prevalence of IPIs found in this study was higher than findings from North Shewa, Ethiopia (17.4%) [12], Debre-Birhan, Northern Ethiopia (9.8%) [24], Dessie, Northern Ethiopia (15.5%) [25], Mozambique (14.5%), Tanzania (15.1%) [26,27], Sub-Saharan Africa (12.1%) [28] and Europe (5.9%) [29]. These variations might be due to differences in hygiene practices, maternal or caregiver educational status, family size (number of under-five-year children), source of water for drinking, diagnostic methods, and socio-economic status of the participants as well as seasonal differences.

Similar studies conducted in two public health facilities, in Hawassa, Southern Ethiopia among under-five-year children present with diarrhea identified the related prevalence of IPIs (26.6%) was found to be positive for at least one Intestinal parasite (IP) species. Double infection was detected to be 3.8%. *E. histolytica/dispar* was the most commonly encountered parasite (11.4%), followed by *G. lamblia* (7%), and 3.8% of children were also positive for the *Cryptosporidium* species. The prevalence of intestinal helminths identified was 8.2% consisting of *Ascaris lumbricoides, Hymenolepsis nana*, and *Trichuris trichiura* [3]. In this study, 11.67% of *E. histolytica/dispar* was identified as slightly higher than in study conducted in certain parts of Ethiopia; Hawassa which was 11.4%, and Debre Birhan, North Shewa which was 5.7% and 5.67% *Giardia lamblia* was also detected. Hence, it was lower than the finding of a similar study conducted in Hawassa two health facilities which were 7%, and that of Debre Birhan, North Shewa was 8.5% where the predominant protozoan parasites were isolated. *Cryptosporidium* species was not detected in our study but it was reported in 3.8% of the children in a study conducted in two health facilities of Hawassa [3]. It might be due to the lack of a detection kit and the absence of molecular technique that makes us miss light infection of Cryptosporidiosis.

Intestinal helminths, particularly soil-transmitted helminths (STHs), were reported commonly infect children in developing countries including Ethiopia [3,12,30]. In this study, helminthic infections were identified in 28 (9.33%) of the children, 11(3.67%) infected with the *A. lumbricoides*, 10(3.33%) *T. trichuria*, 4(1.33%) *H. nana* and 3(1%) children were infected with both *A. lumbricoides* and *T. trichiura*, which is higher than studies conducted in Hawassa two health facilities and Debre Birhan North Shewa Ethiopia, 8.2% and 3.2% respectively. *A. lumbricoides* and *T. trichiura* were the predominant helminthic parasites identified in this study. Studies identified a high prevalence of STHs and *Schistosoma mansoni* in Southwest Ethiopia (76.7%) among elementary school children and recommend urgent deworming of school children according to WHO guidelines [31]. The currently initiated national school-based deworming program might have an impact on the STH infection rate among preschool children in the long run. Since intestinal helminthic infections were acquired from the community from infected sources among all age groups; this deworming program might have a great role in the reduction of the rate of parasitic infection among under-five-years of children. On the other hand, mass treatment didn´t consider drug resistance which might have an impact on the reduced efficacy of albendazole and mebendazole against *A. lumbricoides* and *Hook worms* has been reported in Northwest Ethiopia [3], therefore, deworming might need laboratory confirmation of the species of parasites to be more effective and to minimize to the threat of drug resistance. However, mass treatment (deworming) is important to reduce the rate of intestinal helminthic infection, it´s not as important to protozoan parasitic infections such as *E. histolytica, G. lamblia, C. parvum* this might be taken as a reason why protozoan parasites are the most predominant cause of diarrhea among under-five-year children. In our study only wet mount preparation and formol-ether concentration technique were used to detect the parasites, the number of children infected with parasites could have been underestimated in this study, which might be due to the test method difference, therefore chance of missing light infection is high. In this study, 35 (11.67%) children aged below 2 years were positive for IPs, and 19 (6.33%) children aged 2 up to 3 years were positive whereas the prevalence among those above 3 years was 14(4.67%). This difference is significant with (p-value < 0.05). The higher prevalence of IPs in children aged less than 3 years compared to children aged above 3 years might be due to younger children´s contact with fecally-contaminated soil while playing, they also feed the contaminated soil and their immune status which could predispose them to high risk getting infected.

In this study, 50% of the participants were urban residents, and the prevalence of intestinal parasitic infection among under-five children living in families with less than three members was 18(6%). Therefore, the number of children (family size) was significantly related to the prevalence of IPs confirmed with multivariate analysis (Table 3) might be due to overcrowded living conditions, poverty, and less caregiving than expected. A significant difference in the prevalence of IPs among children from urban areas (6%) and rural areas (16.67%) was observed, this might be due to lack of safe water source, increase soil contact, not washing fruit before a meal, use of unprotected water for drinking and failure to hand washing after defecation. This study also identified that there is a significant association between the educational status of mothers and the prevalence of intestinal parasitic infection, no formal education 27(9%) read and write 19(6.33%), primary 14(4.67%), secondary and above 8(2.67%). Although the knowledge, attitude, and practice towards intestinal parasitic infection were not assessed in this study the prevalence of IPs might be related to the knowledge of guardians on ways of transmission and prevention and control of parasites might play a major role in the epidemiological distribution of the intestinal parasitic infections among under-five-year children.

**Limitation**: the prevalence of IPs among early breastfeeding children and children being breastfed but also receiving complementary food was not assessed in this study. **Recommendation**: in this study high burden of intestinal parasitic infections was recorded among children under five years of age which calls for effective interventional strategies such as deworming (for helminths) and effective anti-protozoal drugs should be considered. Additionally, raising awareness of parents/guardians on keeping personnel hygiene of their children and environmental sanitation is crucial to reduce the burden of parasitic infections, by delivering health educations about intestinal parasites of medical importance. Moreover, strategies targeting the control and prevention of intestinal parasites should be devised by local administrative bodies in the study area.

## Conclusion

The present study identified a high prevalence of intestinal parasitic infections among under- five years of children presenting with diarrhea which is significantly associated with maternal/ guardians´ educational status (no formal education of maternal/ guardians (AOR=94.900, 95% CI: 24.664-365.155), use of unprotected water for drinking (AOR= 25.189, 95% CI: 4.671-135.847), and age group 2-3 years of age children was attributable to IPIs (AOR= 0.466, 95% CI: 0.204,0.976). Other environmental and socio-economic factors play great role to parasitic cause diarrhea among under-five-years of children.

### What is known about this topic

- *Even though diarrheal diseases among under-five-year children are caused by a variety of infectious agents, parasitic etiologies are the major ones;*
- *Identifying the parasitic etiology of diarrhea in general plays the main role in prevention, control and management of the illness; based on environmental condition and personal or behavioral factors, the prevalence and distribution of different parasites from location to location are varied;*
- *Investigating the parasitic etiology of diarrhea in regional as well as local level is paramount to know the profile of those etiologies in need of appropriate interventions*.

### What this study adds

- *In Southwest Ethiopia, diarrhea is one of the most common reasons for children to visit healthcare institutions, however, most of the time, laboratory based investigations of the causative agents of these diarrheal illness is inadequate and most diarrheal cases were managed clinically, hence, parasite detection and identification from diarrhea among under-five-year children were practically not performed in most health facilities of the region;*
- *Identification of different parasitic etiologies of diarrhea among under-five-year children in the study area provides important input to initiate laboratory based diagnosis to be routine;*
- *The finding will be used for health professionals, local, national, and international organizations to give emphasis on taking appropriate measurement and further plan to work on minimizing the burden of diarrheal diseases*.
